# A Bi-Directional Acoustic Micropump Driven by Oscillating Sharp-Edge Structures

**DOI:** 10.3390/mi14040860

**Published:** 2023-04-15

**Authors:** Bendong Liu, Meimei Qiao, Shaohua Zhang, Jiahui Yang

**Affiliations:** 1 Faculty of Materials and Manufacturing, Beijing University of Technology, Beijing 100124, China; qmm1119qx@163.com (M.Q.); zhangshaohua1109@163.com (S.Z.); 2 Beijing Vocational College of Agriculture, Beijing 102208, China; klrs80@sohu.com

**Keywords:** acoustic wave, sharp-edge structure, bi-directional pump, micropump

## Abstract

This paper proposes a bi-directional acoustic micropump driven by two groups of oscillating sharp-edge structures: one group of sharp-edge structures with inclined angles of 60° and a width of 40 μm, and another group with inclined angles of 45° and a width of 25 μm. One of the groups of sharp-edge structures will vibrate under the excitation of the acoustic wave generated with a piezoelectric transducer at its corresponding resonant frequency. When one group of sharp-edge structures vibrates, the microfluid flows from left to right. When the other group of sharp-edge structures vibrates, the microfluid flows in the opposite direction. Some gaps are designed between the sharp-edge structures and the upper surface and the bottom surface of the microchannels, which can reduce the damping between the sharp-edge structures and the microchannels. Actuated with an acoustic wave of a different frequency, the microfluid in the microchannel can be driven bidirectionally by the inclined sharp-edge structures. The experiments show that the acoustic micropump, driven by oscillating sharp-edge structures, can produce a stable flow rate of up to 125 μm/s from left to right, when the transducer was activated at 20.0 kHz. When the transducer was activated at 12.8 kHz, the acoustic micropump can produce a stable flow rate of up to 85 μm/s from right to left. This bi-directional acoustic micropump, driven by oscillating sharp-edge structures, is easy to operate and shows great potential in various applications.

## 1. Introduction

A micropump is an important micro-actuator in MEMS (Micro-Electromechanical Systems), which can realize the directional driving of microfluidics, and is widely used in microsensors, microbiochemical analysis, and various occasions involving microfluid transportation [1]. In recent years, with the rapid development of lab-on-chip technology, it is more urgent to realize the automatic and accurate driving of microfluidics [2,3]. Therefore, the development of the micropump also affects the further integration and performance improvement of microfluidic devices, which is a hot spot in the research of MEMS.

In the past two decades, there has been a surge in studies exploring micropump technologies [4,5,6]. In view of the need for rapid and accurate control of microfluidics in lab-on-chip applications, scholars have explored micropumps with multiple driving modes, including optically-driven pumps [7,8], electro-osmotic pumps [9,10], electrokinetic pumps [11,12], dielectric pumps [13,14], magnetic pumps [15,16], laser-driven pumps [17], pneumatic membrane pumps [18,19,20], bio-hybrid pumps [21,22], and diffuser pumps [23,24]. Recently, the acoustic streaming effect produced by acoustic waves in microfluids has attracted considerable interest, and several microdevices have been explored, including micromixers [25,26,27,28,29], particle manipulation [30,31,32,33,34], and flow control [35,36]. Due to the simple structure, ease of manufacturing, good biocompatibility, fast response, and other acoustic driving characteristics, acoustically oscillating bubbles and acoustically oscillating sharp-edge structures have emerged as promising tools for precise control of the flow in lab-on-chip applications [37]. Admittedly, common problems associated with bubble-based platforms still exist. For example, the size of the bubble in the microchannel could change over time, which could result in an undesired change of the bubble’s resonant frequency for long-term operation [38]. The acoustic streaming generated by the sharp-edge structures excited by the acoustic wave is used to disturb the flow field, and drive the fluid flow in the microchannel, which has the advantages of convenient and stable operation, compared with the acoustic wave-driven microfluid with bubbles. The sharp-edge structures are fabricated on the wall of a microfluidic channel and protrude in the fluid domain. When oscillating in response to acoustic excitation, a sharp-edge structure induces acoustic streaming around its tip [39]. The phenomenon of acoustic streaming is used in a wide variety of microfluidic applications. Zhang et al. investigated experimentally, using particle image velocimetry, the mixing performance of sharp-edge structures at low-frequency acoustic excitation under different experimental conditions (tip angle, vibration amplitude, flow rate). Their results show that the sharper the edge tip, the larger the size of streaming vortices and the spatial extent of the induced streaming, thereby greatly enhancing the mixing efficacy [40]. Experimental investigations by Huang et al. have shown that a sharp-edge-based acoustic micromixer is capable of providing rapid and homogeneous mixing inside a microfluidic channel, and is free from the drawbacks of bubble-based acoustic micromixers [26]. Mohanty showed that vibrating protrusions inside a microchannel are capable of steering fluid away from their relative orientation, which is brought forth by the symmetry-broken design of these protrusions. Additionally, they showed that the flow direction is sensitive to switching acoustic frequencies [41]. The investigation of the acoustic streaming produced by sharp-edge structures is also important for cell and microparticle manipulation and liquid-drop control [42,43,44,45,46].

For the recent micropump based on sharp-edge structures actuated with acoustic waves, the top of the sharp-edge structure is integrated with the top of the microchannel, and the bottom is partially bonded with the glass substrate, due to its small size. When the sharp-edge structure is excited, there is deformation damping between the top of the sharp-edge structure and the top of the microchannel, while there is also some friction between the bottom of the sharp-edge structure and the glass substrate, which reduces the amplitude of the sharp-edge structure, thus affecting the pumping rate. This is different from the existing acoustic-driven micropump with sharp-edge structures as there is a separate gap between the sharp-edge structure and the upper and the lower surfaces of the microchannel, which reduces the damping between the sharp-edge structures and the microchannel, and improves the pumping effect of the micropump. In addition, we propose an acoustic micropump driven by two groups of oscillating sharp-edge structures. The two groups of sharp-edge structures have different inclination angles and widths so they have different resonance frequencies. One of the groups of sharp-edge structures will vibrate under the excitation of the acoustic wave, generated with a piezoelectric transducer, at its corresponding resonant frequency. When one group of sharp-edge structures vibrates, the microfluid flows from left to right. When the other group of sharp-edge structures vibrate, the microfluid flows in the opposite direction, which satisfies the need for selectivity in the direction of pumping.

## 2. Design and Working Mechanism

### 2.1. Design of Acoustic Micropump

**Figure 1:**
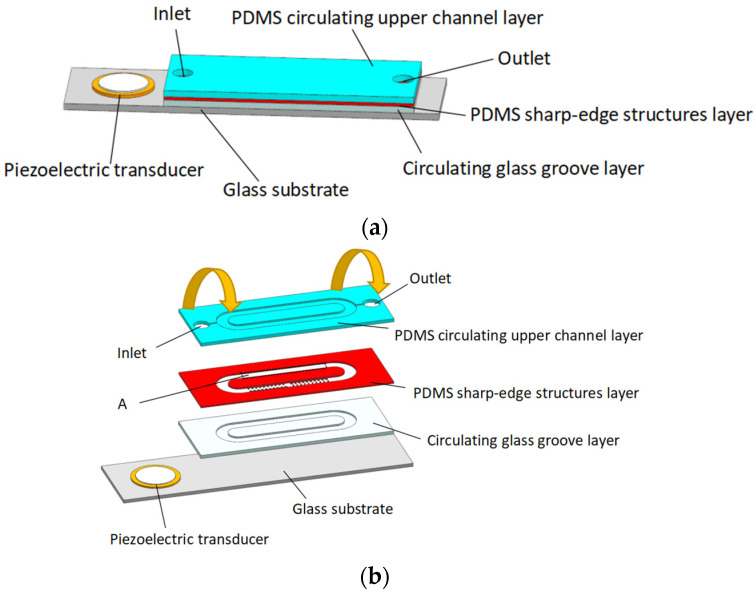
Structure diagram of acoustic micropump: (**a**) Overview of acoustic micropump; (**b**) Exploded view of acoustic micropump.

Figure 1a shows the assembly diagram of the acoustic micropump, which is mainly composed of a piezoelectric transducer, a PDMS circulating upper channel layer, a PDMS sharp-edge structures layer, a circulating glass groove layer, and a glass substrate. Figure 1b is an exploded view of the acoustic micropump structure. The PDMS circulating upper channel layer, the PDMS sharp-edge structures layer, and the circulating glass groove layer are bonded layer by layer to form a sealed microfluidic device with a microchannel. This structure ensures that there are small gaps between the sharp-edge structures and the upper and lower surfaces of the microchannel. The piezoelectric transducer is bonded with the glass substrate with epoxy. The sealed microfluidic device and the glass substrate are bonded together with double-sided tape. When the sharp-edge structure is oscillating, actuated with acoustic waves, there is little deformation damping between the sharp-edge structures and the PDMS circulating upper channel layer, and there is also little friction between the bottom of the sharp-edge structures and the circulating glass groove. As a result of that, the vibration amplitude of the sharp-edge structure is larger, stronger acoustic streaming can be generated, and the pumping effect of the micropump can be improved.

**Figure 2:**
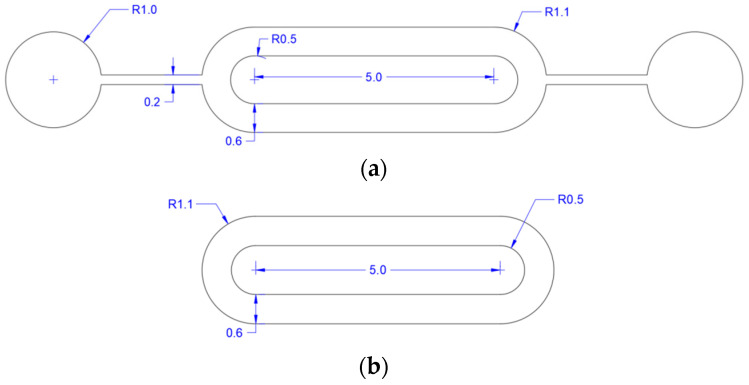
Schematic of PDMS channel and glass groove: (**a**) Dimensions of the PDMS channel; (**b**) Dimensions of the glass groove.

The structural dimensions of PDMS circulating upper channel and circulating glass groove are shown in Figure 2a and Figure 2b, respectively. The structural dimensions of PDMS circulating upper channel are shown in Figure 2a: the width of the pump circulating channel is 600 μm, and the width of the flow channel connecting the pump chamber with the inlet and outlet is 200 μm. The structural dimensions of the circulating glass groove are shown in Figure 2b: the width of the circulating glass groove is 600 μm, and the depth of the circulating glass groove is 25 μm.

**Figure 3:**
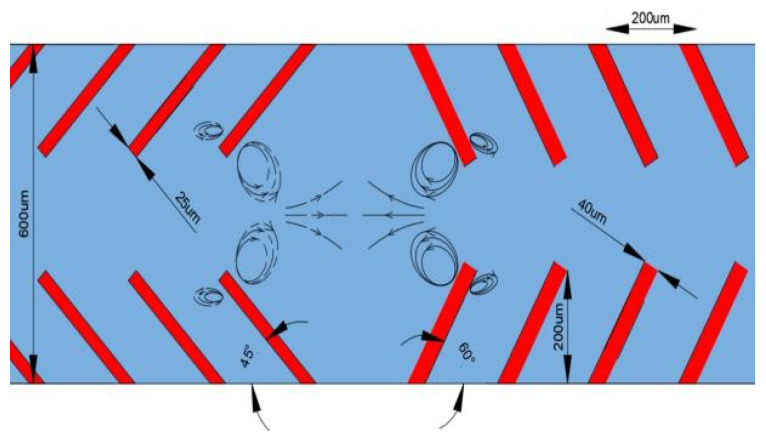
Schematic of PDMS sharp-edge structure layer and working mechanism of acoustic micropump.

The schematic of the PDMS sharp-edge structures layer is shown in Figure 3a, and its structural dimensions are shown in Figure 3b. The total length of the sharp-edge structures layer channel is 7.2 mm, and the thickness of the sharp-edge structures layer is 80 μm. Two groups of sharp-edge structures are designed in the microchannel. One group of the sharp-edge structures is inclined left, with a width of 25 μm and tilt angle of 45°, and the other group of the sharp-edge structures is inclined right, with a width of 40 μm and tilt angle of 60°.

### 2.2. Working Mechanism of Acoustic Micropump

When the piezoelectric transducer is applied with a sinusoidal excitation signal at a resonant frequency, the piezoelectric transducer vibrates and generates acoustic waves. Under the excitation of acoustic waves, the corresponding sharp-edge structures in the micropump are driven to vibrate too. The vibrating sharp-edge structure will generate a pair of asymmetric reverse vortices near its contact with the fluid, which is acoustic streaming [47,48,49], as shown in Figure 3. Actuating with the arrayed acoustic streaming, the fluid in the microchannel can be driven in one selected direction along the microchannel when one group of inclined sharp-edge structures is actuated in the corresponding frequency.

We calculated the dimensionless constant of the Reynolds number to determine whether the fluid flow state in microchannel is laminar or turbulent. The Reynolds number is usually described by the following expression [50]

(1) $$Re=\frac{\rho vd}{\mu }$$

where ρ is the density of the fluid, v is the fluid flow rate, d is the feature size, and μ is the dynamic viscosity of the fluid. When the Reynolds number is greater than 2300, the flow state of the fluid in the pipeline is turbulent, and when it is less than 2300, the flow state is laminar. The Reynolds number in microchannel is calculated to be 150, which means that there is laminar flow in microchannel.

## 3. Fabrication Process of Acoustic Micropump

The fabrication process of the circulating glass groove layer is as follows. First, the positive photoresist was spin-coated on both sides of the cleaned glass sheet, exposed to UV light for one minute, and then developed. After that, the glass sheet was put into a corrosion solution mixed with hydrofluoric acid and ammonium fluoride (1:1.5) at a temperature of 55 °C for 25 min. Finally, the glass was rinsed with acetone to remove the positive photoresist and a groove with a depth of about 25 μm was obtained.

The PDMS circulating upper channel layer was fabricated with standard soft lithography. A layer of SU-8 2050 (Microchem Corp., Westborough, MA, USA) photoresist was spin-coated on a silicon wafer to produce the desired master mold, followed by UV exposure and development. Then, a mixture of a PDMS (Sylgard 184, Dow Corning) with a 10:1 base-to-curing agent ratio was prepared and placed in a vacuum desiccator (Bel-Art Scienceware, Nanjing, China) to remove air bubbles in the mixture. After that, the PDMS mixture was poured onto the master mold and placed on a hot plate to bake for 30 min at 95 °C. Then, the PDMS was peeled off gently, and punched with one inlet and one outlet.

**Figure 4:**
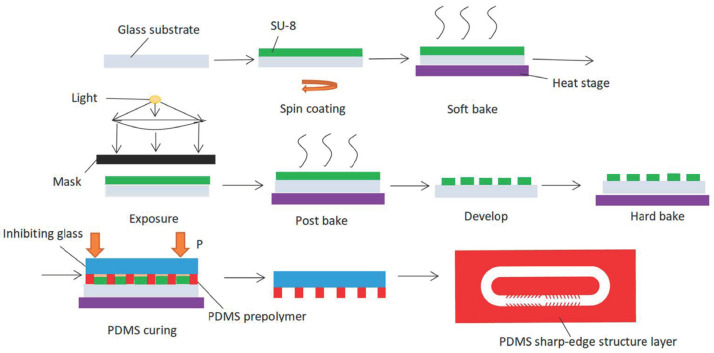
The fabrication scheme of PDMS sharp-edge structure layer (the last image is a top view, and the other images are side views).

The fabrication process of the PDMS sharp-edge structures layer is similar to the fabrication process of the PDMS circulating upper channel layer. The difference is that the sharp-edge structures are a series of separated cantilevers, and there is no polymerized PDMS on the top of the sharp-edge structures. The fabrication process of the desired master mold and the mixture of PDMS with curing agent of the PDMS sharp-edge structures layer is the same as that of the PDMS circulating upper channel layer. Chelating organosilane AATMS((3-(2-aminoethyl)-aminopropyltrimethoxysilane) was used in the fabrication of the PDMS sharp-edge structures layer preventing the PDMS from polymerizing on the top of the sharp-edge structures. A glass slide was immersed in a 6% *w/w* solution of the AATMS molecules in methanol for 1 h, and then baked in an oven at 110 °C for 10 min to covalently link the silane groups to the glass. The glass slide will be used as an inhibiting glass plate in PDMS polymerization. The PDMS prepolymer was poured on the mold and the inhibiting glass plate was placed on top of the liquid prepolymer. After which, a light pressure was applied to planarize the prepolymer layer. The clamping pressure is not critical but must be enough to create a residual prepolymer squeeze film of no more than a few micrometers on top of all protruding features. In our case, we used two clips that exerted about 10 kPa in total on the mold. The prepolymer was then cured in an oven for 30 min at 95 °C. After curing, the thin squeeze film of prepolymer on top of the protruding mold features remains unpolymerized while the bulk PDMS polymerized. The formation of residual film between PDMS sharp-edge structures was also prevented, by inhibiting PDMS polymerization on the protruding part of the mold. The process flow diagram is shown as Figure 4:

The PDMS sharp-edge structures layer, attached to the inhibiting glass plate, bonded with the circulating glass groove layer after plasma treatment. The inhibiting glass plate was removed by gently shearing it off from the PDMS sharp-edge structures film. The PDMS circulating upper channel layer was aligned and reversibly bonded with the PDMS sharp-edge structures layer with a microscope, forming a sealed microfluidic device.

**Figure 5:**
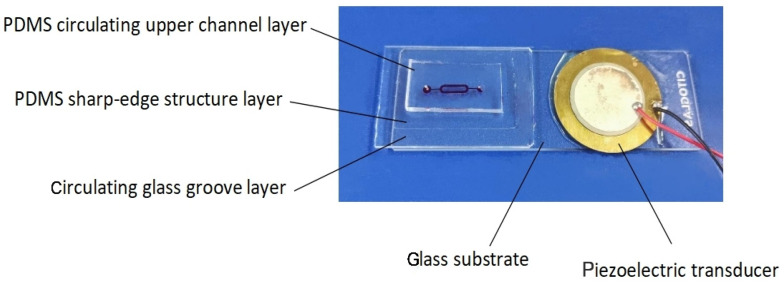
Photograph of the acoustic micropump.

A piezoelectric transducer (SY-27T-3.5A1, Yaoze Electronics, Shenzhen, China) and the glass substrate were bonded together with AB epoxy (DP460, 3M, Maplewood, MN, USA), and the sealed microfluidic device and the glass substrate were bonded together, with double-sided tape (9080, 3M, Maplewood, MN, USA). A photograph of the acoustic micropump is shown in Figure 5.

## 4. Results and Discussion

### 4.1. Composition of Experimental System

**Figure 6:**
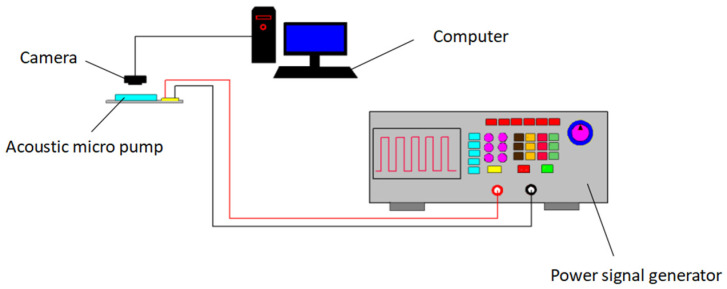
Schematic diagram of experimental test system.

The experimental test system is shown in Figure 6. There was a CCD camera (YH5001-3, Shanghai Optical Instrument Factory, Shanghai, China) and a power signal generator (ATG-2042, Agitek, Xi’an Antai Test Equipment Co., Ltd., Xi’an, China). The CCD camera was mounted on a microscope (6XD-3, Shanghai Yongheng Optical Instrument Manufacturing Co., Ltd., Shanghai, China) and connected to a computer, which was used to record the images.

### 4.2. Working Frequency of Acoustic Micropump

**Figure 7:**
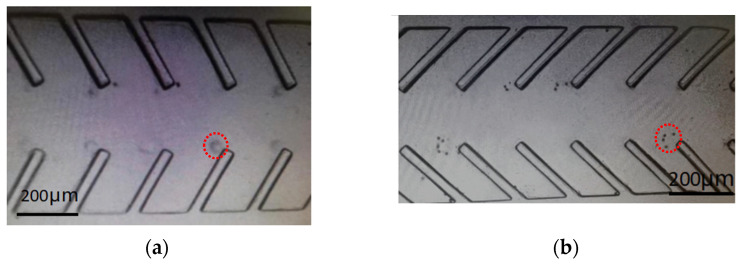
The microscopy images of acoustic streaming around sharp-edge structures: (**a**) Acoustic streaming around sharp-edge structures with an inclined angle of 60° at 20.0 kHz; (**b**) Acoustic streaming around sharp-edge structures with an inclined angle of 45° at 12.8 kHz.

Some deionized water mixed with polystyrene beads, with a diameter of 5 μm, was injected into the micropump. The polystyrene beads were used to characterize the acoustic streaming. We injected the mixed solution into the micropump, and after the solution had stabilized, sinusoidal excitation signal was applied to the piezoelectric transducer connected to the power signal generator. We considered the frequency at which the acoustic streaming phenomenon is most obvious, and the microbead in the micropump moved faster, as the resonance frequencies of the micropump. To determine the resonance frequencies corresponding to different directions of motion, by sweeping the frequency with a 100 Hz increment from 1 kHz to 30 kHz, we observed that the acoustic streaming patterns were most visible around the tips of the oscillating sharp-edge structures with an inclined angle of 60° when the piezoelectric transducer was activated at 20.0 kHz; when the applied frequency is around 20 kHz, the movement speed of the microbeads significantly slowed down, or even stopped. Therefore, we set 20.0 kHz as the resonant frequency of the sharp-edge structure with an inclined angle of 60° Similarly, obvious acoustic streaming patterns were developed around the tips of the oscillating sharp-edge structures with an inclined angle of 45° when the piezoelectric transducer was activated at a frequency of 12.8 kHz. The microscopy images of acoustic streaming at resonance frequency around sharp-edge structures are shown in Figure 7.

### 4.3. Pumping Rate of Acoustic Micropump

**Figure 8:**
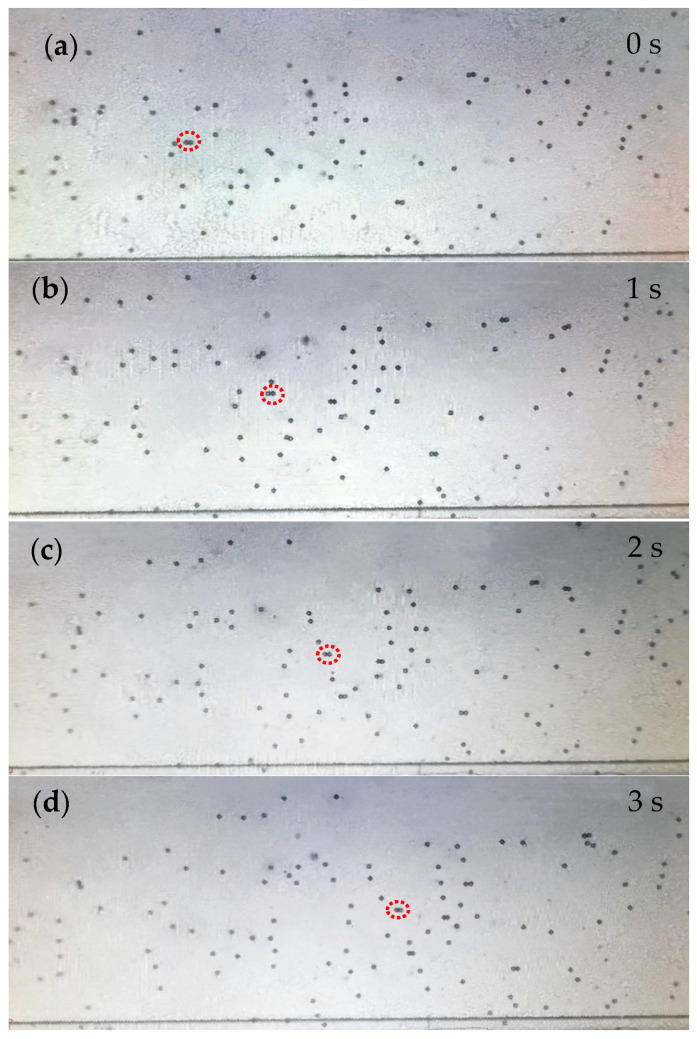
Images showing the pumping behavior at 20.0 kHz by indicating the movement of polystyrene beads in acoustic micropump at different time frames when (**a**) t = 0 s, (**b**) t = 1 s, (**c**) t = 2 s, (**d**) t = 3 s.

In order to visualize the pumping flow, we used polystyrene beads with a diameter of 5 μm, and polystyrene beads with a diameter of 10 μm, to characterize the speed in the micropump, respectively. During the experiment, we found that the movement of beads with a diameter of 5 μm under the microscope was not as clear as that of beads with a diameter of 10 μm, so the speed of the beads with a diameter of 10 μm was considered as flow speed in our experiment. Deionized water solution, with polystyrene beads with a diameter of 10 μm, was injected into the micropump. According to the experiments of frequency selection, the working frequency for the group sharp-edge structure with an angle of 60° is 20.0 kHz and the working frequency for the group sharp-edge structure with angle of 45° is 12.8 kHz. During the experiment, a sinusoidal excitation signal was applied to the piezoelectric transducer through a signal generator at resonant frequency, while the input voltage was adjusted to observe the movement of the beads in the micropump. We used a camera connected to a computer to capture the motion state of the polystyrene beads in the micropump, then selected appropriate time points as the starting point and end point, and measured the motion distance of the polystyrene beads during this period on the computer, to calculate the motion speed of the polystyrene beads. Figure 8 shows the movement of polystyrene beads in the micropump within 3 s when the piezoelectric transducer was excited with 200 Vpp at a frequency of 20.0 kHz. The polystyrene beads (circled in red) moved from left to right (Video S1). The pumping flow rate could be calculated by multiplying the cross-sectional area of the channel with the flow speed. The micropump flow rate was about 15 mL/min when the pumping rate was about 125 μm/s. The images in Figure 8 were captured in domain A that was shown in Figure 1b.

In order to verify that the micropump with a certain gap between the sharp-edge structures and the upper and lower surfaces of the microchannel has a higher pumping speed than the sharp-edge structures bonded on the substrate and directly connected on the upper surface of the microchannel, a mold with sharp-edge structures and a circular microchannel with the same dimension as shown in Figure 3 was fabricated. A PDMS copy with the same dimension, sharp-edges, and microchannel was obtained with the mold using casting and curing process, and then directly bonded with a glass substrate forming a micropump. The result shows that the maximum pumping rate is about 25 μm/s, actuated with a peak voltage of 200 Vpp, at various frequencies.

**Figure 9:**
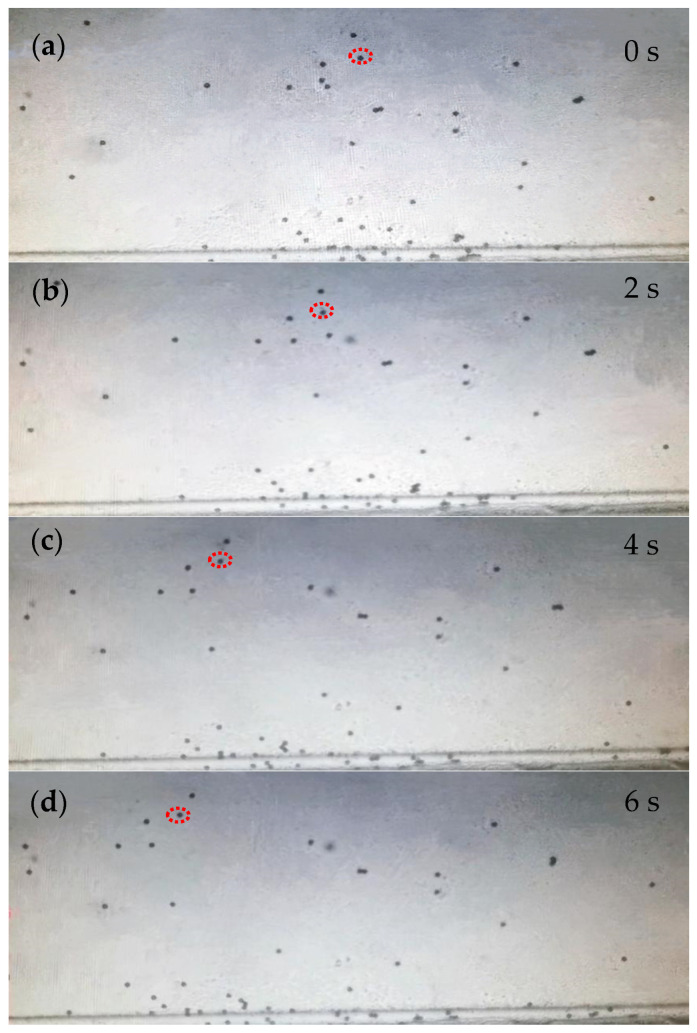
Images showing the pumping behavior at 12.8 kHz by indicating the movement of polystyrene beads in acoustic micropump at different time frames when (**a**) t = 0 s, (**b**) t = 2 s, (**c**) t = 4 s, (**d**) t = 6 s.

Figure 9 shows the movement of polystyrene beads in the micropump when the piezoelectric transducer was excited with 200 Vpp at frequency of 12.8 kHz, the polystyrene beads (circled in red) moved from right to left (Video S2) and the pumping rate was about 85 μm/s. Similarly, the micropump flow was calculated to be approximately 10 mL/min. The images were also captured in domain A, as shown in Figure 1b.

**Figure 10:**
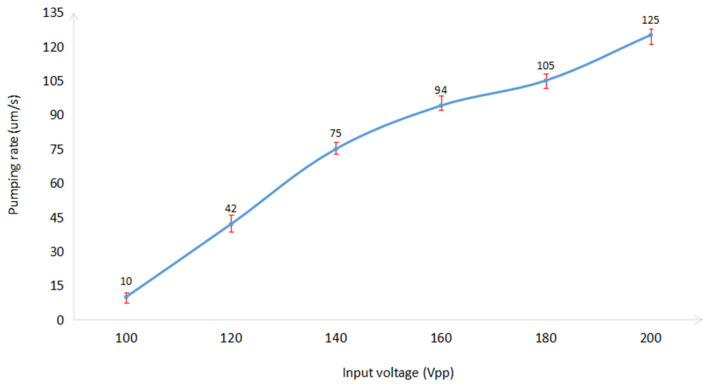
Pumping flow rates with various voltages applied to the piezoelectric transducer activated at 20.0 kHz.

As shown in Figure 10, the relationship between the micropump pumping flow rate and the voltage was obtained by adjusting the applied voltage. It was observed that the acoustic streaming begins to occur around the sharp-edge structure of 60° in the micropump, when the piezoelectric transducer was activated at 20.0 kHz, with the applied voltage of 100 Vpp. The polystyrene beads in the micropump start to move at a speed of 10 μm/s (the micropump flow is approximately 0.12 mL/min) with an applied voltage of 100 Vpp. As the input voltage increases, the velocity of the beads gradually increases. When the input voltage increased to 200 Vpp, our micropump pumping rate reaches 125 μm/s (the micropump flow is approximately 15 mL/min).

**Figure 11:**
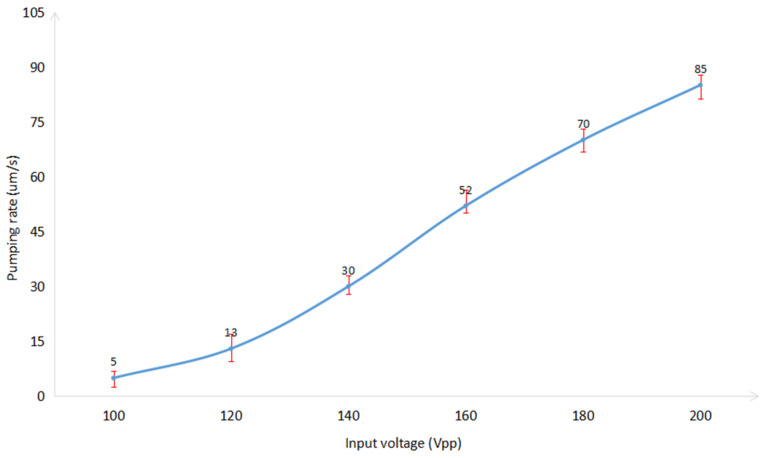
Pumping flow rates with various voltages applied to the piezoelectric transducer activated at 12.8 kHz.

The relationship of the pumping flow rates with various applied voltages, activated at 12.8 kHz, is shown in Figure 11. The pumping flow rate reaches 85 μm/s (the micropump flow is approximately 10 mL/min) when the input voltage increases to 200 Vpp.

During the experiment, we considered that a portion of acoustic waves during the propagation of microfluidics would be converted from mechanical energy to thermal energy, resulting in an increase in the temperature of the fluid in the microchannel. In the future, we will analyze the temperature changes of the microfluidics in the micropump during operation, and design relevant tests to analyze the biocompatibility of the micropump.

## 5. Conclusions

This paper presents a bi-directional acoustic micropump driven by two groups of oscillating titled sharp-edge structures with different widths and different tilt angles, which can achieve the selection of pumping direction. In addition, by improving the manufacturing process of the micropump, the cantilevered sharp-edge structures in the microchannel were designed, which can reduce the damping of sharp-edge structures in the channel. Through experimental comparison, this cantilevered sharp-edge structure has a better driving effect in an acoustic micropump. The pumping rate is about 125 μm/s (the micropump flow is approximately 15 mL/min) from left to right, actuated with a voltage of 200 Vpp, at a frequency of 20.0 kHz. The pumping rate is about 85 μm/s (the micropump flow is approximately 10 mL/min) from right to left, actuated with a voltage of 200 Vpp, at a frequency of 12.8 kHz. The pumping rate in the micropump can also be adjusted by adjusting the input voltage. The micropump in this paper not only has advantages in simplicity, stability, reliability, cost-effectiveness, controllability, and flexibility, but also has great value in many laboratory chip applications when combined. Microfluidic directional driving devices with sharp-edge structures, excited with acoustic waves, have potential biomedical applications, including on-chip laboratory, portable or implantable drug delivery devices.

## Data Availability

The data that support the findings of this study are available from the corresponding authors upon request.

## Supplementary Materials

The following supporting information can be downloaded at: https://www.mdpi.com/article/10.3390/mi14040860/s1, Video S1: The movement of polystyrene beads in acoustic micropump when the piezoelectric transducer was excited with 200 Vpp at a frequency of 20.0 kHz. Video S2: The movement of polystyrene beads in acoustic micropump when the piezoelectric transducer was excited with 200 Vpp at a frequency of 12.8 kHz.
